# The oral mucosal microbiome in children with Crohn’s disease exhibits reduced biodiversity compared to healthy children, revealed by 16s profiling

**DOI:** 10.1080/20002297.2017.1325254

**Published:** 2017-06-09

**Authors:** Khalid Elmaghrawy

**Affiliations:** ^a^ Trinity College, University of Dublin, Dublin, Ireland

## Abstract

The oral microbiome was examined in a cohort of treatment naïve children diagnosed with Crohn’s disease (n=27, CD) or ulcerative colitis (n=6, UC). A cohort of 28 children were grouped as a healthy control (HC) group. Bacterial DNA was extracted from tongue and buccal swabs and the V1-V2 region of the 16s gene was amplified and sequenced using the MiSeq. Sequences were analysed with the Mothur pipeline.

Reduced biodiversity of the tongue was indicated by differences in the inverse Simpson’s index for both sites (CD tongue=9.39; HC=12.87). Analysis of species richness by rarefaction showed a significant reduction in species richness in CD tongue samples compared to HC tongue samples. Analysis of community structure and membership using AMOVA showed that the populations on HC and CD tongues were significantly different (P <0.001). LEfSe analysis identified 20 OTUs that were significantly enriched on the tongues of healthy children including *H. parainfluenzae, N. flavescens, F. periodonticum, Streptococcus sp., Porphyromonas sp., Actinomyces sp*. Children with Crohn’s have an altered microflora that may contribute to their oral health problems. These data could potentially be used to diagnose a patients overall gastrointestinal health.

